# Modulation of GABA_A_ receptors and of GABAergic synapses by the natural alkaloid gelsemine

**DOI:** 10.3389/fnmol.2022.1083189

**Published:** 2023-01-17

**Authors:** Ana M. Marileo, Javiera Gavilán, Victoria P. San Martín, Cesar O. Lara, Anggelo Sazo, Carola Muñoz-Montesino, Patricio A. Castro, Carlos F. Burgos, Elías Leiva-Salcedo, Luis G. Aguayo, Gustavo Moraga-Cid, Jorge Fuentealba, Gonzalo E. Yévenes

**Affiliations:** ^1^Department of Physiology, Faculty of Biological Sciences, Universidad de Concepción, Concepción, Chile; ^2^Millennium Nucleus for the Study of Pain (MiNuSPain), Santiago, Chile; ^3^Department of Biology, Faculty of Chemistry and Biology, Universidad de Santiago de Chile, Santiago, Chile

**Keywords:** GABA_A_ receptor, analgesia, anxiolysis, natural alkaloid, gelsemine, toxicology

## Abstract

The *Gelsemium elegans* plant preparations have shown beneficial activity against common diseases, including chronic pain and anxiety. Nevertheless, their clinical uses are limited by their toxicity. Gelsemine, one of the most abundant alkaloids in the *Gelsemium* plants, have replicated these therapeutic and toxic actions in experimental behavioral models. However, the molecular targets underlying these biological effects remain unclear. The behavioral activity profile of gelsemine suggests the involvement of GABA_A_ receptors (GABA_A_Rs), which are the main biological targets of benzodiazepines (BDZs), a group of drugs with anxiolytic, hypnotic, and analgesic properties. Here, we aim to define the modulation of GABA_A_Rs by gelsemine, with a special focus on the subtypes involved in the BDZ actions. The gelsemine actions were determined by electrophysiological recordings of recombinant GABA_A_Rs expressed in HEK293 cells, and of native receptors in cortical neurons. Gelsemine inhibited the agonist-evoked currents of recombinant and native receptors. The functional inhibition was not associated with the BDZ binding site. We determined in addition that gelsemine diminished the frequency of GABAergic synaptic events, likely through a presynaptic modulation. Our findings establish gelsemine as a negative modulator of GABA_A_Rs and of GABAergic synaptic function. These pharmacological features discard direct anxiolytic or analgesic actions of gelsemine through GABA_A_Rs but support a role of GABA_A_Rs on the alkaloid induced toxicity. On the other hand, the presynaptic effects of the alkaloid provide an additional mechanism to explain their beneficial effects. Collectively, our results contribute novel information to improve understanding of gelsemine actions in the mammalian nervous system.

## 1. Introduction

*Gelsemium elegans* extracts have been used to treat various common diseases, including neuralgia, sciatica, rheumatoid arthritis, chronic pain, and anxiety (Rujjanawate et al., 2003; Dutt et al., 2010; Jin et al., 2014; Lin et al., 2021). Nevertheless, *Gelsemium elegans* preparations have been also classified as strong poisons and its clinical uses are limited by their intrinsic toxicity, which may include body tremors, dyspnea, convulsions, tremors, respiratory arrest, and even death (Zhou et al., 2017; Lin et al., 2021; Qu et al., 2021; Yang et al., 2022). Interestingly, both the beneficial and toxic effects of *Gelsemium elegans* extracts in humans have been reproduced in experimental models (Dutt et al., 2010; Jin et al., 2014; Lin et al., 2021). Purified alkaloids from *Gelsemium* plants have replicated several of the actions shown by extracts. Gelsemine, one of the most abundant alkaloids in *Gelsemium elegans*, has shown analgesic and anxiolytic effects on murine behavioral models (Liu et al., 2013; Meyer et al., 2013; Zhang et al., 2013; Zhang and Wang, 2015) and improved sleep instabilities in mice with neuropathic pain (Wu et al., 2015).

The molecular targets underlying the therapeutic and toxic effects of gelsemine remain unclear. Several studies have reported that some of the beneficial effects of gelsemine are dependent on mechanisms involving the modulation of the enzyme 3α-hydroxysteroid oxide-reductase (3α-HSOR) (Venard et al., 2008; Shoaib et al., 2019) and of glycine receptors (GlyRs) (Lara et al., 2016; Zeilhofer et al., 2021). The proposed mechanism to explain analgesic and anxiolytic actions of gelsemine involves the GlyR-triggered stimulation of the spinal neurosteroid production through 3α-HSOR, which in turn potentiates the activity of spinal GABA_A_ receptors (GABA_A_Rs), generating anxiolysis and analgesia (Shoaib et al., 2019). On the other hand, the gelsemine induced toxicity has been related with the functional inhibition of GlyRs (Lara et al., 2016). Despite its potential relevance as a gelsemine target, potential direct actions of the alkaloid on GABA_A_Rs and on GABAergic synapses have been minimally studied. Since GABA_A_Rs are the main biological targets of benzodiazepines (BDZs), the mainstay group of drugs used to treat anxiety and sleep disorders, and of other clinically relevant drugs, a potentiation of GABA_A_Rs by gelsemine has been considered as a potential mechanism of action (Ghit et al., 2021; Knoflach and Bertrand, 2021). In addition, the analgesic effects of classical BDZs (Ralvenius et al., 2015) suggest a contribution of gelsemine to pain control through GABA_A_Rs. BDZ sensitive GABA_A_Rs are composed of γ2 and α1, α2, α3 or α5 subunits, mostly together with β2 or β3 subunits. Classical BDZs bind to the interface between γ2 and the mentioned α subunits at the extracellular domain of the receptor to potentiate the GABA-activated currents. Conversely, a negative modulation of GABA_A_Rs by the alkaloid may contribute to its toxicity. In line with this notion, a recent study showed that the toxicity induced by gelsenicine, another *Gelsemium* alkaloid, was significantly reverted by the systemic application of diazepam, a classical BDZ, to rats (Li et al., 2022). Therefore, using electrophysiological recordings of recombinant and native receptors, here we intend to define whether a direct modulation of GABA_A_Rs by gelsemine exists, Due to their relevance for the effects of BDZs and, in some cases, due to their wide expression at GABAergic synapses, we focus on recombinant γ2-containing GABA_A_Rs and on the impact of the alkaloid on native GABA_A_Rs and on GABAergic synapses.

## 2. Materials and methods

### 2.1. Chemicals

Gelsemine hydrochloride (>99% purity) was obtained from ChemFaces (Wuhan, PRC). All other chemicals were purchased from Tocris (Bristol, UK), Hello-Bio (Bristol, UK), Sigma-Aldrich (St. Louis, MO, USA) or AK Scientific (Union City, CA, USA).

### 2.2. Cell culture, plasmids, and transfection

HEK293 cells (CRL-1573; ATCC, VA, USA) were cultured using standard methods (Lara et al., 2016). The cells were transfected with GABA_A_R α subunits (1, 2, 3, 5), α1(H101R), β(2,3) and γ2 expressing plasmids using Lipofectamine (ThermoFisher, Carlsbad, CA, USA). The plasmids encoding the rat α and the β subunits subcloned in the pRK5 vector (Benson et al., 1998; Paul et al., 2014) and the rat γ2 subunit subcloned in the pIRES2-EGFP (Moraga-Cid et al., 2011) were used along the study, using a transfection ratio for of 1:1:5 of α:β:γ2 encoding plasmids.

### 2.3. Animals and cortical cultures

All animal care and experimental protocols of this study were conducted in accordance with the ethical protocols established by the National Institutes of Health (NIH, USA) and were supervised and approved by the Bioethical Committee of the University of Concepcion. The animal studies were reported as recommended by the ARRIVE guidelines (McGrath and Lilley, 2015). A total of 15 animals were used in this study. The animals were treated humanely with due consideration to the alleviation of distress and discomfort. Cultured cortical neurons were prepared as previously described (Aguayo and Pancetti, 1994; Zemoura et al., 2013). Additional details are available on the Supplementary information.

### 2.4. Electrophysiology

GABA-evoked currents were recorded from transfected HEK293 cells and from cultured neurons in the whole-cell voltage-clamp configuration at room temperature (20–24°C) using a holding potential of −60 mV (Paul et al., 2014). In brief, patch electrodes (3–4 MΩ) were pulled from borosilicate glasses and were filled with internal solution, which contains (in mM): 120 CsCl, 8 EGTA, 10 HEPES (pH 7.4), 4 MgCl2, 0.5 GTP and 2 ATP. The external solution contained (in mM) 140 NaCl, 5.4 KCl, 2.0 CaCl2, 1.0 MgCl2, 10 HEPES (pH 7.4) and 10 glucose. Whole-cell recordings were performed with an Axoclamp 200B (Molecular Devices, Sunnyvale, CA, USA) or with a HEKA EPC-10 (HEKA Elektronik GmbH, Germany) amplifiers and were acquired using Clampex 10.1 or Patch Master software. Data analysis was performed off-line using Clampfit 10.1 (Axon Instruments, Sunnyvale, CA, USA) and MiniAnalysis 6.0.3 (Synaptosoft, CA, USA). Exogenous GABA-evoked currents were obtained using a manually applied pulse (3–4 s) of the agonist and an outlet tube (200 μm ID) of a gravity-fed micro perfusion system. The EC_10-15_ values for the recombinant and neuronal receptors were obtained experimentally after the successive application of increasing GABA concentrations (0.01–300 μM). Further details are available in the Supplementary information.

### 2.5. Statistical analyses

Normality was verified using the Shapiro–Wilk or D’Angostino Pearson tests depending on sample sizes. All results are presented as mean ± SEM. Statistical analysis and graphs were done with Origin (version 6.0–8.0) and GraphPad Prism 6. Values of *p* < 0.05, *p* < 0.001, and *p* < 0.0001 were considered statistically different. Statistical comparisons were performed using paired Student *t*-tests, unpaired Student *t*-tests with Welch’s correction and ANOVA followed by Bonferroni post-hoc test. Statistical comparisons of cumulative probability distributions were performed using the Kolmogorov–Smirnov (K-S) test.

## 3. Results

### 3.1. Modulation of recombinant GABA_A_Rs by gelsemine

**Figure 1:**
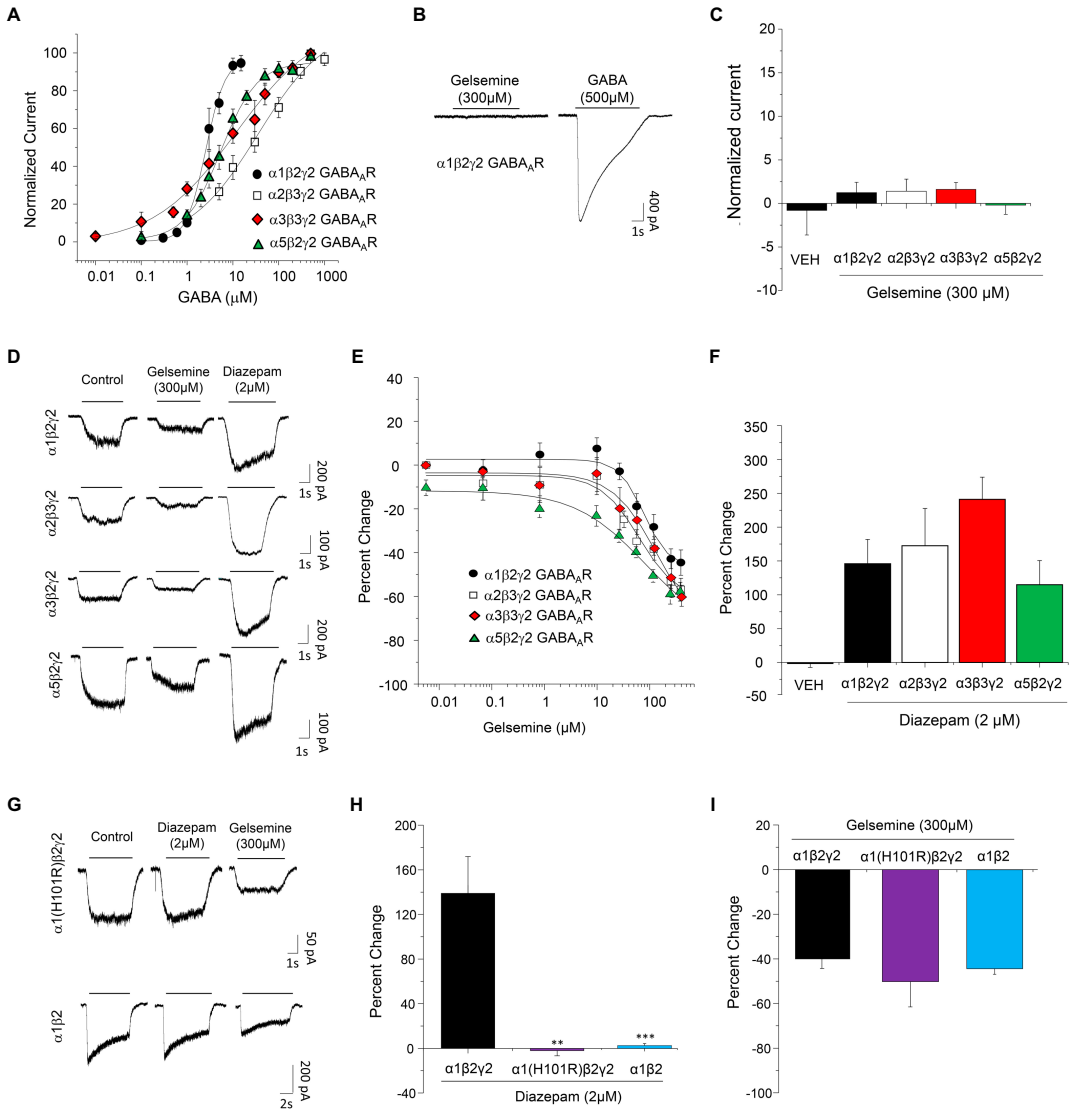
Modulation of recombinant BDZ-sensitive GABA_A_Rs by gelsemine. **(A)** Concentration-response curves to GABA of α1β2γ2, α2β3γ2, α3β3γ2, and α5β2γ2 GABA_A_Rs expressed in HEK293. Data points are means ± SEM from 6–8 cells. **(B)** Representative traces illustrating the actions of gelsemine in the absence of GABA on α1β2γ2 GABA_A_Rs. **(C)** The graph summarizes the lack of detectable agonist effects of gelsemine on α1β2γ2, α2β3γ2, α3β3γ2, and α5β2γ2 GABA_A_Rs. Data are means ± SEM of 5–6 cells. **(D)** Representative traces showing the inhibitory effects of gelsemine (300 μM) and the positive effects of diazepam (2 μM) on GABA-evoked currents through α1β2γ2, α2β3γ2, α3β3γ2, and α5β2γ2 GABA_A_Rs. **(E)** The graph summarizes the effects of different gelsemine concentrations (0.1–300 μM) on the GABA-evoked currents of cells expressing α1β2γ2, α2β3γ2, α3β3γ2, and α5β2γ2 GABA_A_Rs. **(F)** The graph shows the effects of diazepam (2 μM) on the GABA-evoked currents. Data are means ± SEM of 5–10 cells. **(G)** Representative traces illustrating the effects of diazepam (2 μM) and gelsemine (300 μM) on GABA-evoked currents through α1(H101R)β2γ2 and α1β2 GABA_A_Rs. **(H,I)** The graphs summarize the diazepam and gelsemine actions on α1(H101R)β2γ2 and α1β2 GABA_A_Rs. For panel **(H)**, ***p < 0.001, **p < 0.01, ANOVA followed by Bonferroni post-hoc test, *F*(2,14) = 3,860 . For panel **(I)**, differences were not significant. ANOVA followed by Bonferroni post-hoc test, *F*(2,14) = 3.440.

To determine whether gelsemine may behave as a positive allosteric modulator of GABA_A_Rs, we examined the effects of gelsemine on the BDZ-sensitive GABA_A_Rs configurations expressed on mammalian synapses (Fritschy, 2015; Ralvenius et al., 2015; Knoflach and Bertrand, 2021) using whole-cell patch clamp recordings. We first determined the GABA sensitivity of recombinant α1β2γ2, α2β3γ2, α3β3γ2 and α5β2γ2 expressed on HEK 293 cells (Table 1; Figure 1A). Although several of these values resemble those obtained in previous studies (Paul et al., 2014; Sallard et al., 2021), we found a Number of Hill (n_H_) value for α1β2γ2 receptors close to 2, which is higher than reported values (Benson et al., 1998; Mortensen et al., 2012). The n_H_ is commonly used in pharmacology as a description of ligand cooperativity, and n_H_ values higher than 1 implies a positive cooperative binding. These differences may arise from the expression system, receptor heterogeneity, post-translational modifications, or fast desensitization (Sallard et al., 2021). We next evaluated the potential direct effects of gelsemine on GABA_A_Rs in the absence of its natural agonist, GABA. The application of gelsemine (300 μM) in the absence of GABA did not elicit any change on the holding currents, indicating the lack of agonistic activity [α1β2γ2; 1.2 ± 1.2% (*n* = 6), α2β2γ2; 1.4 ± 1.3% (*n* = 6), α3β3γ2; 1.6 ± 0.8% (*n* = 6) and α5β2γ2; −0.2 ± 1.0 (*n* = 6)] (Figures 1B,C). We then assessed the effects of gelsemine (0.01–300 μM) on the GABA-activated currents activated by a sub-saturating agonist concentration (i.e., EC_10-15_) (Figures 1D,E). Micromolar concentrations of the alkaloid (>10–25 μM) significantly inhibited the GABA-evoked chloride currents in a concentration-dependent manner. The analysis of concentration-response curves to gelsemine revealed a similar profile of inhibition for the five GABA_A_R subtypes studied (Figure 1E; Table 1). None of the GABA_A_R subtypes studied were fully blocked by the alkaloid.

**Table 1:** Sensitivity to GABA and gelsemine of recombinant and native GABA_A_Rs.

	GABA sensitivity				Gelsemine sensitivity			
GABA_A_R	EC_50_ (μM)	n_H_	I_max_(pA)	N	IC_50_ (μM)	n_H_	Maximal modulation (%)	*n*
α1β2γ2	3.2 ± 0.2	1.8 ± 0.2	3,792 ± 257	6	66.8 ± 16.0	1.9 ± 0.7	−44.3 ± 9.9	6
α2β3γ2	29.8 ± 10.1	0.6 ± 0.1	1,958 ± 371	7	55.2 ± 13.7	1.2 ± 0.5	−56.5 ± 4.9	6
α3β3γ2	12.4 ± 5.5	0.5 ± 0.1	2,227 ± 475	6	55.6 ± 12.1	1.2 ± 0.5	−63.5 ± 5.0	10
α5β2γ2	5.4 ± 0.3	1.1 ± 0.1	1,428 ± 302	8	72.1 ± 7.9	0.7 ± 0.3	−61.1 ± 3.8	6
α1(H101R)β2γ2	2.3 ± 0.1	1.5 ± 0.2	1,946 ± 266	5	–	–	–	–
α1β2	2.1 ± 0.1	1.6 ± 0.2	1,641 ± 271	6	–	–	–	–
Native	2.1 ± 0.5	1.0 ± 0.1	2,492 ± 568	12	89.1 ± 16.4	1.2 ± 0.1	−49.6 ± 4.3	8
+ Gels.	7.4 ± 1.0*	1.2 ± 0.2	1,995 ± 434	12	–	–	–	–

Values of the concentration–response curve parameters (EC_50_ and Hill coefficients, nH) were obtained from the curve fits of normalized concentration–response data points from the indicated number of cells. The maximal current modulation was obtained using 300 μM of gelsemine. Results are presented as mean ± SEM. *p < 0.01, unpaired Student *t*-test, native versus gelsemine treated (+ Gels., 200 μM).

As expected from previous reports (Paul et al., 2014; Ralvenius et al., 2015), diazepam (2 μM) was able to potentiate the function of the five GABA_A_R subtypes studied [α1β2γ2; 146.1 ± 35.9% (*n* = 6), α2β2γ2; 172.6 ± 54.9% (*n* = 6), α3β3γ2; 242.1 ± 32.8% (*n* = 10) and α5β2γ2; 115.0 ± 35.6 (*n* = 6) (Figure 1F)], confirming the correct expression of GABA_A_Rs of 2α-2β-1γ2 stoichiometry. The negative modulation of the GABAergic currents exerted by gelsemine on these receptor subtypes suggest that its mechanism of action is distinct than the prototypical BDZ, diazepam. However, the alkaloid still may exert its actions on the receptor by inverse agonism on the BDZ site or through molecular sites unrelated to γ2 subunits (Olsen et al., 2019; Ghit et al., 2021; Kim and Hibbs, 2021). Consequently, we evaluated the effects of gelsemine on GABA_A_Rs composed of α1(H101R)β2γ2 and of α1β2 subunits. The point-mutated α1GABA_A_R subunit contains a histidine-to-arginine mutation at the position 101 which disrupts the BDZ binding site, generating GABA_A_Rs insensitive to diazepam but normally sensitive to the agonist GABA (Benson et al., 1998; Ralvenius et al., 2015; Knoflach and Bertrand, 2021). We select the α1β2γ2 subtype to assess the relevance of the BDZ binding site because is the most widely expressed GABA_A_R subtype in the brain (Fritschy, 2015). On the other hand, α1β2 receptors are not sensitive to BDZs due to the absence of the α1-γ2 interphase, but conserve the sensitivity to its agonist, GABA, and to other modulators, such as propofol, neurosteroids and etomidate (Olsen et al., 2019; Kim and Hibbs, 2021; Knoflach and Bertrand, 2021). Our experiments demonstrated that α1(H101R)β2γ2 and α1β2 GABA_A_Rs were insensitive to diazepam 2 μM (−2.0 ± 4.7% and 4.0 ± 2.9%, respectively) in comparison with wild-type α1β2γ2 (146.1 ± 35.9%) (Figures 1G,H). Conversely, gelsemine elicited a similar inhibition of the GABA-evoked currents in these receptors (α1(H101R)β2γ2: −50.8 ± 11.8%, α1β2: −44.1 ± 2.5% and wild-type α1β2γ2: −40.1 ± 5.4%) (Figures 1G,I).

### 3.2. Gelsemine modulation of native GABA_A_Rs expressed in cortical neurons

**Figure 2:**
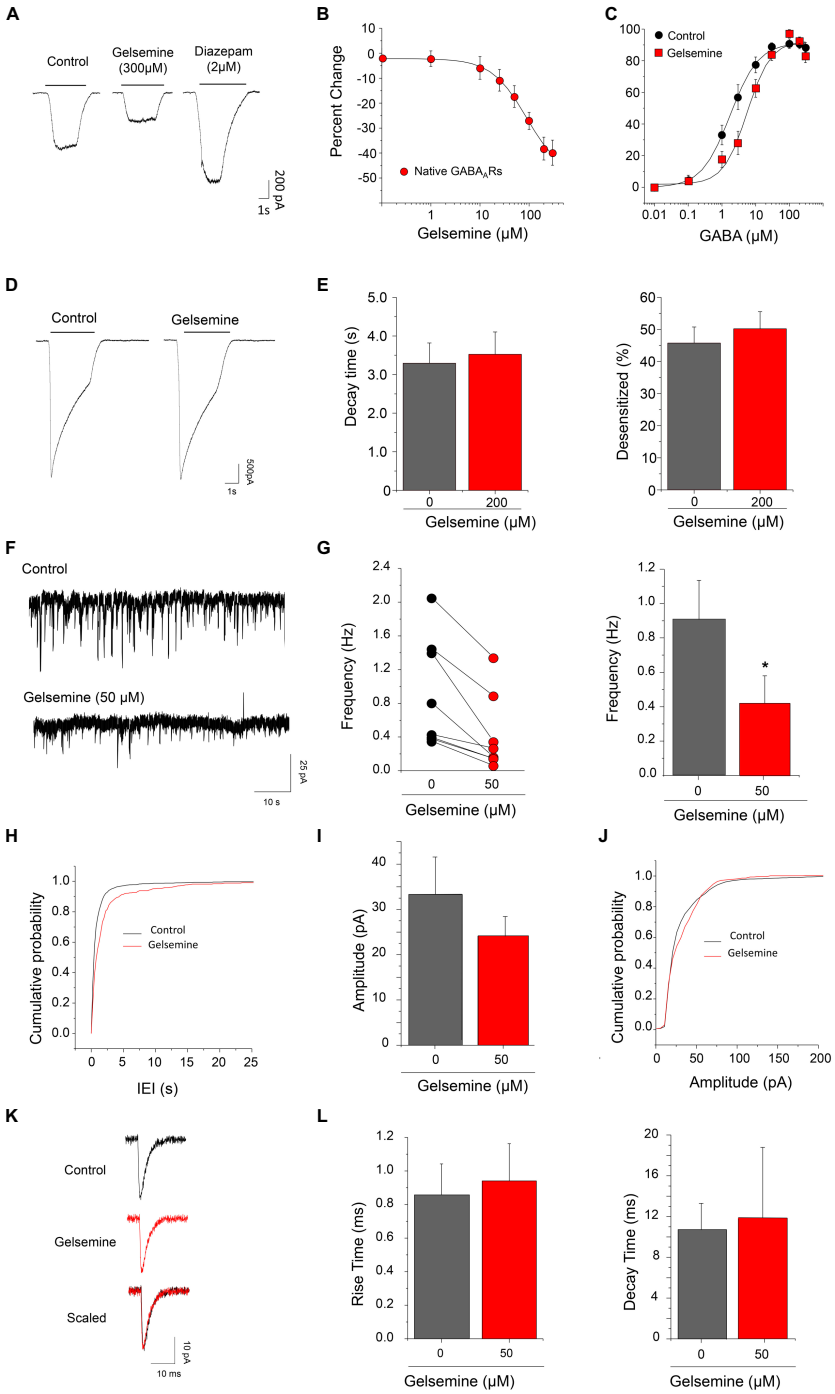
Gelsemine modulation of native GABA_A_Rs and of GABAergic neurotransmission. **(A)** Representative traces showing the inhibitory effects of gelsemine (300 μM) and the positive effects of diazepam (2 μM) on GABA-evoked currents (0.5–1 μM) through native GABA_A_Rs. **(B)** The graph summarizes the gelsemine effects (0.1–300 μM) on the GABA-evoked currents. Data are means ± SEM of 8 neurons. **(C)** GABA concentration-response curves in the absence (black circles) and presence (red squares) of gelsemine (200 μM). Data are means ± SEM of 12 cells per condition. The GABA sensitivity was significantly altered by the alkaloid (Table 1). **(D)** Representative current traces from native GABA_A_Rs using a saturating concentration of GABA (300 μM) in the absence and presence of gelsemine (200 μM). **(E)** The graphs summarize the effects of gelsemine on the decay time (left panel) and the percentage of desensitized current (right panel) of the GABA-evoked currents. Differences were not significant (*n* = 12, per condition). **(F)** Examples of current traces showing the GABAergic synaptic activity before and during the application of 50 μM gelsemine. **(G)** The scatter graph and the bar plot depicts the effect of 50 μM of gelsemine on the mIPSCs frequency in 8 neurons. The average frequency of GABAergic mIPSCs was significantly diminished by gelsemine. *p < 0.05, paired Student *t*-test. **(H)** Cumulative probability distribution of the inter-event intervals of GABAergic mIPSCs in the absence or the presence of 50 μM of gelsemine. The distribution was significantly altered by the alkaloid. *p* < 0.0001; Kolmogorov–Smirnoff test. **(I)** The plot summarizes the effects of gelsemine on the average amplitude. Differences were not significant. *p* = 0.15, paired Student *t*-test. **(J)** Cumulative probability of the amplitudes of GABAergic mIPSCs in the absence or the presence of gelsemine. Differences were not significant (Kolmogorov–Smirnoff test). **(K)** Average GABAergic current traces before and during the application of 50 μM of gelsemine. **(L)** The bar plots summarize the effects of gelsemine on the rise time and on the decay time of GABAergic mIPSCs. Differences were not significant.

To assess the modulation of native GABA_A_Rs by gelsemine, we carried out electrophysiological experiments on mouse cultured cortical neurons. At the point of our electrophysiological experiments (i.e., 9–12 days in culture), these preparations consist of a heterogeneous population of neurons and glial cells. Prior evaluating the gelsemine sensitivity, we assess the expression of BDZ-sensitive GABA_A_Rs using RT-qPCR assays and electrophysiological experiments. Our results show that these cultures expressed the necessary subunit repertoire to configure functional GABA_A_Rs sensitive to BDZs (Supplementary Figure S1; Ralvenius et al., 2015; Knoflach and Bertrand, 2021). In line with these data, the GABA-evoked currents obtained from these neurons were sensitive to diazepam (64.6 ± 10.9%, *n* = 7, 2 μM diazepam. Figure 2A).

We next evaluated the effect of different concentrations of gelsemine (0.1–300 μM) on GABA-evoked currents using sub-saturating concentration of agonist (0.5–1 μM). The chloride currents through native GABA_A_Rs were inhibited by gelsemine in a concentration dependent fashion (Figures 2A,B; Table 1). The IC_50_ (89.1 ± 16.4 μM) and the maximal inhibition value (−49.6 ± 4.3% with 300 μM) of the gelsemine-induced inhibition obtained from these neurons were comparable with the results obtained from recombinant receptors (Figure 1; Table 1). We then analyzed whether gelsemine may affect the GABA sensitivity or the maximal GABA-evoked current. Analysis of concentration-response curves showed that gelsemine (200 μM) significantly decreased the apparent affinity for GABA of neuronal receptors, shifting the average EC_50_ value from 2.1 ± 0.5 μM to 7.41 ± 1.0 μM [*t*(8.9) = 4.714, **, *p* = 0.0011, unpaired Student *t*-test] (Figure 2C; Table 1). On the other hand, the alkaloid did not significantly affect the maximal current amplitude elicited by saturating concentrations of GABA (Figure 2D; Table 1). Although these data suggest that gelsemine modulates GABA_A_Rs by decreasing the apparent affinity for GABA, the alkaloid may still influence the desensitization rates. To evaluate this possibility, we next examined the kinetic properties of the macroscopic GABA-activated currents stimulated by a saturating agonist concentration (300 μM). These analyses indicated that gelsemine did not modify the fraction of desensitized current or the decay time constant of the GABA-evoked currents (Figures 2D,E).

### 3.3. Gelsemine effects on the synaptic function of cultured cortical neurons

In previous studies, it has been described that gelsemine is able to diminish the frequency of glycinergic spontaneous miniature post-synaptic currents of spinal neurons, without modify the average amplitude (Lara et al., 2016). Whether GABAergic synapses are also modulated by gelsemine is unknown. Therefore, we analyzed GABAergic spontaneous miniature post-synaptic currents (mIPSCs) on cortical neurons. Although cultured cortical neurons do not display glycinergic synaptic events due to the absence of synaptically-released glycine (Zeilhofer et al., 2021), they express functional GlyRs that could be still activated or modulated through tonic or presynaptic mechanisms (Siebler et al., 1993; Avila et al., 2013; McCracken et al., 2017). Considering this information, we included strychnine in all our experiments to avoid any potential glycinergic actions of gelsemine. Under these conditions, the application of the GABAergic antagonist bicuculline completely blocked the detection of synaptic currents, confirming their GABAergic nature.

**Figure 3:**
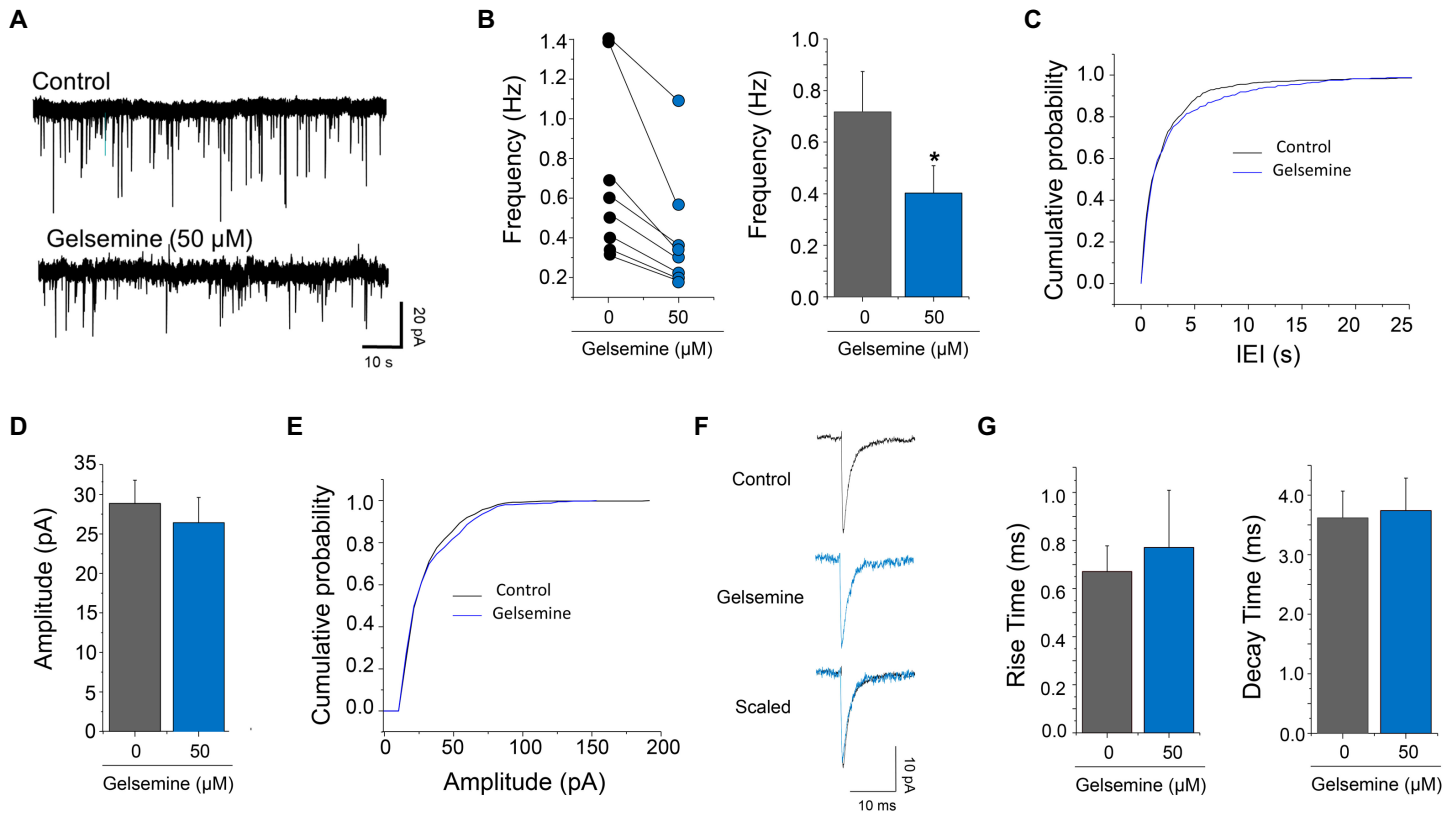
Actions of gelsemine on glutamatergic neurotransmission. **(A)** Examples of current traces showing the glutamatergic synaptic activity before and during the application of 50 μM gelsemine. **(B)** The scatter graph and the bar plot depict the effect of 50 μM of gelsemine on the frequency of synaptic events in 8 neurons. The average frequency of glutamatergic mEPSC was significantly diminished by gelsemine. *p < 0.05, paired Student *t*-test. **(C)** Cumulative probability distribution of the inter-event intervals of mEPSCs in the absence or the presence of 50 μM of gelsemine. The distribution was significantly altered by the alkaloid. *p* < 0.0001; Kolmogorov–Smirnoff test. **(D)** The bar plot summarizes the effects of gelsemine on the amplitude. The amplitude was not significantly diminished by gelsemine. *p* = 0.98, paired Student *t*-test. **(E)** Cumulative probability distribution of the amplitudes of mEPSCs in the absence or the presence of 50 μM of gelsemine. Differences were not significant (Kolmogorov–Smirnoff test). **(F)** Average glutamatergic current traces before and during the application of 50 μM of gelsemine. **(G)** The bar plot summarizes the effects of gelsemine on rise time and decay time of glutamatergic mEPSCs. Differences were not significant.

The GABAergic mIPSCs from cultured cortical neurons displayed an average frequency of 0.91 ± 0.23 Hz (*n* = 8). The application of 50 μM of gelsemine reduced the frequency of GABAergic mIPSCs in all the neurons recorded (Figures 2F,G). Gelsemine decreased the average frequency to a value of 0.42 ± 0.16 Hz (*n* = 8) (*p* < 0.01, paired Student *t*-test vs. control condition) (Figure 2G) and altered the cumulative probability of the inter-event intervals (*p < 0.0001; K-S test) (Figure 2H). Despite the reduction of the frequency, all the neurons still displayed several mIPSCs in the presence of the alkaloid. The remaining GABAergic synaptic events showed an unaltered average amplitude (33.3 ± 8.2 pA to 24.8 ± 4.3 pA, *p* = 0.15, paired Student *t*-test) (Figures 2I,J; Supplementary Table S1). Likewise, the average rise time and the mean decay time kinetics of GABAergic mIPSCs were not modified by the alkaloid (Figures 2K,L; Supplementary Table S1). To assess whether the alkaloid is active as a modulator of excitatory neurotransmission, we next evaluated glutamatergic miniature excitatory post-synaptic currents (mEPSCs) in the presence of 50 μM of gelsemine (Figure 3; Supplementary Table S1). To avoid any gelsemine-mediated effects related with the modulation of GlyRs or GABA_A_Rs, the mEPSCs were pharmacologically isolated by the application of TTX together with strychnine and bicuculline. Our results showed that the application of 50 μM gelsemine significantly diminished the frequency of mEPSCs in all the neurons recorded (Figures 3A,B). The average frequency (Control = 0.72 ± 0.16 Hz; gelsemine = 0.40 ± 0.11 Hz, *n* = 8; p < 0.01, paired Student *t*-test) (Figure 3B) and the cumulative probability distribution of the inter-event intervals were also significantly reduced by the alkaloid (*p < 0.0001; K-S test) (Figure 3C). Nevertheless, gelsemine did not modify the average amplitude (28.8 ± 2.9 pA to 26.4 ± 3.1 pA, *p* = 0.98, paired Student *t*-test) nor the rise or decay time parameters of glutamatergic mEPSCs (Figures 3D–G; Supplementary Table S1). Further experiments showed that the application of 100 μM of gelsemine abolished the presence of detectable GABAergic and glutamatergic events (not shown).

## 4. Discussion

The GABAergic neurotransmission is the main inhibitory control of the central nervous system. The activation of GABA_A_Rs regulates critical neurophysiological processes, such as anxiety, muscle tone, memory, pain, among others (Fritschy, 2015; Ghit et al., 2021; Knoflach and Bertrand, 2021). In line with its relevance, the GABA_A_Rs pharmacology has been widely developed (Ghit et al., 2021; Knoflach and Bertrand, 2021; Cerne et al., 2022). GABAergic ligands include a large variety of agonists, antagonists, and allosteric modulators coming from diverse origins, including compounds purified from fungi and plants, as well as endogenous molecules and molecules of synthetic design. Therefore, different GABA_A_R subtypes possesses many acceptor sites for these modulators, which can be located in both intra-subunit and inter-subunit sites within the transmembrane or the extracellular domains of the ion channel complex (Olsen et al., 2019; Kim and Hibbs, 2021). Here, we characterize the modulation of recombinant and native GABA_A_Rs by gelsemine, a natural *Gelsemium* alkaloid. Our results indicate that gelsemine is a negative modulator, but not an agonist, of BDZ-sensitive GABA_A_Rs. Native GABA_A_Rs expressed in cultured cortical neurons were also inhibited by gelsemine. The frequency of spontaneous miniature GABAergic and glutamatergic synaptic currents was significantly diminished by the alkaloid.

A previous study reported that native GABA_A_Rs expressed in cultured spinal neurons were inhibited by gelsemine (Lara et al., 2016). Our results using recombinant receptors of defined composition showed that gelsemine inhibited the GABA-activated chloride currents through GABA_A_Rs configurations that are widely expressed in the CNS and that have been associated with the actions of BDZs (Ghit et al., 2021; Knoflach and Bertrand, 2021; Cerne et al., 2022). The inhibitory actions exerted by gelsemine on these GABA_A_Rs were similar, implying the absence of subunit-selective effects. In line with these observations, native GABA_A_Rs expressed in cultured cortical neurons, which are also BDZ-sensitive, were inhibited by gelsemine. Despite the heterogeneity of GABA_A_R configurations expressed on these neurons, the IC_50_ and the maximal current inhibition were comparable with the results from recombinant GABA_A_Rs. In a wider context, the results reported here allow us to generate a more complete profile of the gelsemine actions on the two inhibitory neurotransmitter-gated ion channels, GABA_A_Rs and GlyRs. Previous findings reported an average IC_50_ values of ≈40 ± 4 μM and a maximal percentage of inhibition ≈ −87 ± 5% (applying 300 μM gelsemine) for recombinant GlyRs (Lara et al., 2016). Thus, a comparison of the experimental IC_50_ values and the maximal inhibition of both chloride-permeable channels establish that GlyRs are more sensitive to the gelsemine inhibition than GABA_A_Rs. Although we cannot discard other mechanisms, we think that these evidences contribute to provide, at least in part, the neurophysiological bases of the toxicity of gelsemine and of *Gelsemium elegans* extracts (Lin et al., 2021). The direct inhibitory actions of gelsemine on GABAergic and glycinergic currents correlate well with a general depression of the inhibitory control of the central nervous system and with the symptomatology of these alkaloids in mammals. In agreement with this concept, a recent study in rats found that diazepam reverted the toxicity induced by gelsenicine, another *Gelsemium* alkaloid (Li et al., 2022). Further studies on inhibitory receptors may generate a rational framework to design targeted pharmacological approaches to treat acute *Gelsemium* alkaloid toxicity through, for example, positive allosteric modulators of GlyRs or of GABA_A_Rs (Lara et al., 2020; Cerne et al., 2022).

The molecular mechanisms underlying the gelsemine inhibition of GABA_A_Rs are unknown. Previous studies have shown that gelsemine increased the EC_50_ for glycine of recombinant GlyRs (Lara et al., 2016). Additional reports showed that gelsemine displaced the binding of ^3^H-strychnine on spinal cord tissue (Shoaib et al., 2019). Likewise, our results showed that the gelsemine significantly decreased the apparent agonist sensitivity of native GABA_A_Rs without significant changes on the maximal currents or on the desensitization rates. Although these data collectively suggest that gelsemine may act as competitive antagonist on both ion channels, molecular or structural corroborations of the gelsemine binding to the orthosteric site of GABA_A_Rs or GlyRs has been not reported yet. Our results however have shown that the negative gelsemine effects on GABA_A_Rs require only α and β subunits, discarding the participation of γ2 subunits and hinting at a potential binding to the orthosteric site. Nevertheless, GABA_A_Rs composed of α and β subunits still have modulatory sites for other ligands (e.g., propofol, neurosteroids, among others) (Olsen et al., 2019; Kim and Hibbs, 2021) and, therefore, the actions of gelsemine through these sites cannot be discarded.

Our analysis of the regulation of GABAergic synapses by gelsemine revealed additional biological actions. Our findings showed that gelsemine significantly decreased the frequency of GABAergic mIPSCs and glutamatergic mEPSCs without changes on the average amplitude nor the kinetics of the synaptic currents, suggesting the participation of a presynaptic target. Based on previous studies (Lara et al., 2016) and the data presented here, one can argue that gelsemine exerted the synaptic modulation through presynaptic inhibitory receptors. The relevance of presynaptic chloride-permeable ion channels has been demonstrated in previous reports (Jang et al., 2006; Schicker et al., 2008; Choi et al., 2013; McCracken et al., 2017). Nevertheless, a role of presynaptic GABA_A_Rs or GlyRs on the gelsemine actions is not supported by several lines of evidence. First, our electrophysiological analyses were performed under the presence of strychnine. Second, the isolation of glutamatergic events was done using bicuculline and strychnine. Third, the gelsemine concentration used to assess the synaptic effects (i.e., 50–100 μM) will not be able to generate a strong modulation of post-synaptic GABA_A_Rs. Altogether, these observations suggest that gelsemine reduces excitatory and inhibitory transmission by a mechanism not related with inhibitory ion channels. In this context, it is feasible that the function of voltage-gated calcium channels, presynaptic GPCRs, or other proteins involved in the presynaptic calcium regulation could be directly affected by the alkaloid (Zhu and Pan, 2005; Pernía-Andrade et al., 2009; Booker et al., 2020). Further experiments of intracellular calcium dynamics may help to define the molecular nature of additional gelsemine-sensitive synaptic proteins in future projects.

Whether our results help to clarify the medicinal effects of gelsemine and of *Gelsemium* elegans extracts in mammals is complex to determine. Gelsemine concentrations within the nanomolar range have been reported to stimulate the production of spinal neurosteroids and/or to produce analgesia or anxiolysis (Shoaib et al., 2019). Since the direct effects of gelsemine on GABA_A_Rs occurs using concentrations >10–25 μM, a contribution of this type of fast modulation to the beneficial effects is unlikely. Thus, we believe that our findings discard that the anxiolytic (Liu et al., 2013; Meyer et al., 2013), hypnotic (Wu et al., 2015) and analgesic effects (Zhang et al., 2013; Zhang and Wang, 2015; Shoaib et al., 2019) of gelsemine are mediated by the direct potentiation of BDZ-sensitive GABA_A_Rs. On the other hand, the regulation of the excitatory and inhibitory synaptic activity mediated by gelsemine may contribute to explain some of its therapeutic actions. Presynaptic attenuation of synaptic activity is a common mechanism of action of clinically relevant drugs (Taylor et al., 2007; Alles et al., 2020) and a fast reduction of neurotransmission may contribute to control neural circuits implicated in anxiety and pain. In this context, it should be noted that the time scales of the beneficial behavioral effects of *Gelsemium* alkaloids have shown a degree of variability. For example, Venard et al. (2008)) showed that gelsemine enhanced the spinal neurosteroid production after 3 h incubation. Analgesic effects have been detected 10 min after the gelsemine intrathecal injection (Zhang et al., 2013), while anxiolytic effects have been reported after 30 min of subcutaneous administration or after 7 consecutive days of treatment with the alkaloid (Liu et al., 2013; Meyer et al., 2013). Therefore, we believe that further research on the fast effects of gelsemine at the synaptic level will complement the proposed indirect mechanisms centered on the gelsemine-induced stimulation of neurosteroids production and the subsequent modulation of GABA_A_Rs, contributing to generate a broader and more detailed mechanistic representation of the gelsemine actions on the CNS.

In summary, our findings establish gelsemine as a negative modulator of the GABA_A_Rs subtypes that contribute to the therapeutic BDZ effects. Thus, the functional pharmacological profile of gelsemine reject direct anxiolytic and analgesic actions through BDZ-sensitive GABA_A_Rs. However, the reduction of GABAergic currents elicited by gelsemine, together with the previously reported GlyR inhibition (Lara et al., 2016), may explain a significant part of the toxicity profile of gelsemine in mammals. On the other hand, our results showed that the alkaloid diminished the GABAergic synaptic function likely through a presynaptic mechanism. These presynaptic actions of the alkaloid may provide a complementary hypothesis to explain its beneficial effects. Future studies focused on the presynaptic compartment will help to further understand the fast actions elicited by gelsemine on the mammalian central nervous system.

## Data availability statement

The raw data supporting the conclusions of this article will be made available by the authors, without undue reservation.

## Author contributions

AM, JG, VS, CL, AS, CB, PC, and EL-S performed the experiments and analyzed the results. JF, CM-M, LA, and GM-C contribute with reagents, critical equipment, and general resources. GM-C, JF, EL-S, and GY conceived and designed the study, analyzed the data, and wrote the article. All authors have read and approved the final manuscript.

## Funding

This work was supported by ANID-FONDECYT 1211082 (to GY), ANID-FONDECYT 1211095 (GM-C), ANID-FONDECYT 1200908 (to JF) and ANID-FONDECYT 1220680 (to EL-S). This work was also supported by the Millennium Nucleus for the Study of Pain (MiNuSPain). MiNuSPain is a Millennium Nucleus supported by the Millennium Science Initiative NCN19_038 of the Ministry of Science, Technology, Knowledge and Innovation, Chile. AM, JG, VS, AS, and CL were supported by ANID-CONICYT doctoral fellowships. AS was also supported by the Graduate School Fellowship of the University of Concepción (MSc in Neurobiology).

## Conflict of interest

The authors declare that the research was conducted in the absence of any commercial or financial relationships that could be construed as a potential conflict of interest.

## Publisher’s note

All claims expressed in this article are solely those of the authors and do not necessarily represent those of their affiliated organizations, or those of the publisher, the editors and the reviewers. Any product that may be evaluated in this article, or claim that may be made by its manufacturer, is not guaranteed or endorsed by the publisher.
