# Supplementary material for: Modulation of GABAA receptors and of GABAergic synapses by the natural alkaloid gelsemine
Source: Front Mol Neurosci. 2023 Jan 17;15:1083189. doi: 10.3389/fnmol.2022.1083189 (PMC9887029; doi:10.3389/fnmol.2022.1083189)
Supplement: Supplementary file 1 [file Data_Sheet_1.docx]

**Supplementary information**

**1. MATERIALS AND METHODS**

*1.1. Animals and cortical cultures.*

All animal care and experimental protocols of this study were conducted in accordance with the ethical protocols established by the National Institutes of Health (NIH, USA) and were supervised and approved by the Bioethical Committee of the University of Concepcion. The animal study protocol was approved by the Ethics Committee of the University of Concepcion (protocol code 1170252 approved in 01.06.2017 and CEBB944-2021 approved in 28.05.2021). The animal studies were reported as recommended by the ARRIVE guidelines (McGrath and Lilley, 2015). A total of 15 animals were used in this study. The animals were treated humanely with due consideration to the alleviation of distress and discomfort. Cultured cortical neurons were prepared as previously described (Aguayo and Pancetti, 1994; Zemoura et al., 2013). The electrophysiological experiments were performed after 9–12 days of culture. In brief, a pregnant mouse (C57BL/6J) was placed in a closed bucket with isoflurane before cervical dislocation. The cortical area from five–six mouse embryos (E18.5) was plated at a confluence of 320.000 cells/mL onto 18 mm glass coverslips coated with poly-L-lysine (70–150 kDa; Trevigen, MD, USA). The feeding medium consisted of 90% minimal essential medium (GIBCO, MD, USA), 5% heat inactivated horse serum (Hyclone, UT USA), 5% FBS (GIBCO, MD, USA), and a mixture of nutrient supplements.

1.2*. Electrophysiology.*

GABA-evoked currents were recorded from transfected HEK293 cells and from cultured cortical neurons in the whole-cell voltage-clamp configuration at room temperature (20–24°C) using a holding potential of -60 mV (Paul et al., 2014; Lara et al., 2016). Patch electrodes (3–4 MΩ) were pulled from borosilicate glasses and were filled with internal solution, which contains (in mM): 120 CsCl, 8 EGTA, 10 HEPES (pH 7.4), 4 MgCl2, 0.5 GTP and 2 ATP. The external solution contained (in mM) 140 NaCl, 5.4 KCl, 2.0 CaCl2, 1.0 MgCl2, 10 HEPES (pH 7.4) and 10 glucose. Whole-cell recordings were performed with an Axoclamp 200B (Molecular Devices, Sunnyvale, CA, USA) or with a HEKA EPC-10 (HEKA Elektronik GmbH, Germany) amplifiers and were acquired using Clampex 10.1 or Patch Master software. Data analysis was performed off-line using Clampfit 10.1 (Axon Instruments, Sunnyvale, CA, USA) and MiniAnalysis 6.0.3 (Synaptosoft, CA, USA). Exogenous GABA-evoked currents were obtained using a manually applied pulse (3–4 s) of the agonist and an outlet tube (200 μm ID) of a gravity-fed micro perfusion system. Stock solutions of gelsemine were prepared in high purity distilled water and subsequently diluted into the recording solution on the day of the experiment. The EC_10-15_ values for the recombinant and neuronal receptors were obtained experimentally after the successive application of increasing concentrations of GABA (0.01 - 300 μM). The normalized current was calculated using the equation Normalized current =100 × (I / Imax). The concentration-response curve parameters (EC_50_ and n_H_) were obtained from the fitted curve of normalized concentration-response data points to the equation I_agonist_ = I_max_ (agonist)_nH_ / ((agonist)_nH_ + (EC_50_) _nH_). I_agonist_ is the current in the presence of a given sub saturating concentration of GABA, nH is the Hill coefficient, EC_50_ is the concentration required for half-maximal response, Imax is the maximum amplitude of the current. The potential agonistic effects of gelsemine were assessed using 300 µM of the alkaloid without GABA. These results were normalized with the current evoked by a saturating GABA concentration (500 µM). The effects of diazepam and gelsemine on GABA evoked currents were obtained using a co-application of sub-saturating GABA concentration (GABA EC_10-15_) together with the modulator, without a pre-application procedure. The percent change was calculated using the equation Percent change=100× ((I_drug_ −I_GABA_)/I_GABA_), where I_drug_ is the current in the presence of a given concentration of drug (diazepam or gelsemine), and I_GABA_ is the amplitude of the control GABA current (EC_10-15_). A rapid solution exchanger method was used for the desensitization experiments (Warner Instruments, USA). The percentage of desensitizing current and the decay time constant of GABA_A_Rs in the absence or in the presence of gelsemine were obtained from whole-cell current traces of 5 s duration. GABAergic miniature inhibitory postsynaptic currents (mIPSCs) in cortical neurons were isolated using strychnine (2 μM), CNQX (4 μM), AP5 (50 μM) and TTX (0.3 μM). The remaining mIPSCs were fully blocked by bicuculline (5 μM). The glutamatergic miniature excitatory postsynaptic currents (mEPSCs) were recorded in the presence of extracellular TTX (0.3 μM), bicuculline (5 μM) and strychnine (2 μM). Under these conditions, all the remaining synaptic events were blocked by a cocktail of CNQX (4 μM) and AP5 (50 μM). The rise and decay time was fitted to a single exponential function and was calculated off-line using Clampfit 10.1 or MiniAnalysis 6.0.3.

**Table S1.** Effects of gelsemine on miniature GABAergic mIPSCs and glutamatergic mEPSCs from cultured cortical neurons.

|  | Frequency (Hz) | Amplitude (pA) | Rise Time (ms) | Decay time (ms) | n | N |
| --- | --- | --- | --- | --- | --- | --- |
| mIPSCs | 0.91± 0.23 | 33.3± 8.2 | 0.86±0.18 | 10.72±2.62 | 8 | 912 |
| + Gelsemine | 0.42±0.16* | 24.8± 4.3 | 0.94±0.22 | 11.79±6.92 | 8 | 538 |
| mEPSCs | 0.72±0.16 | 28.8± 2.9 | 0.67± 0.11 | 3.62±0.45 | 8 | 1724 |
| + Gelsemine | 0.40±0.11* | 26.4±3.1 | 0.77±0.24 | 3.74±0.55 | 8 | 912 |

*, P<0.05, paired Student t-test. A concentration of 50 μM of gelsemine was used. N, number of events analyzed, n, number of cells. A concentration of 100 μM of gelsemine abolished both the excitatory and inhibitory synaptic activity (n=3-4 neurons per condition, N=0).


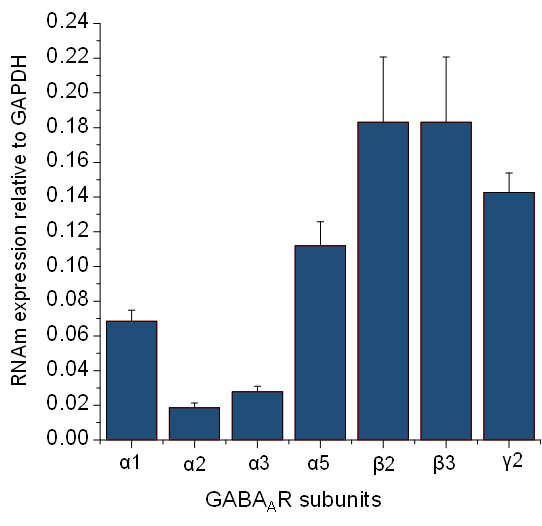


**Figure S1.** **Quantitative reverse transcriptase PCR analysis of BDZ-sensitive GABAAR subunits in cultured cortical neurons.** The graph summarizes the expression of subunits α1, α2, α3, α5, β2, β3, γ2 relative to GADPH. For the retro-transcription of total RNA to cDNA, the Affinity-Script qPCR cDNA Synthesis kit (Agilent technologies) was used. To perform the qPCR, the Brilliant II SYBR® Green QPCR Master Mix kit (Agilent technologies) and the Mx3000P qPCR System (Agilent technologies) were used. The primers for GABAARs subunits have been previously described by Linnemann et al. 2006 and Szemes et al. 2013. The primers for GADPH enzyme were used as a control (Forstera et al., 2016). N=3.
